# Comparison of Cost Analysis in Patients with Tetrahydrobiopterin-Responsive and Non-Responsive Phenylketonuria in Turkey

**DOI:** 10.3390/nu16101444

**Published:** 2024-05-10

**Authors:** Merve Karaca Sahin, Ayse Cigdem Aktuglu Zeybek, Tanyel Zubarioglu, Mehmet Serif Cansever, Ertugrul Kıykım

**Affiliations:** 1 Department of Pediatrics, Cerrahpasa Medical Faculty, Istanbul University-Cerrahpasa, 34098 Istanbul, Turkey; karacasahinmerve@gmail.com; 2 Division of Nutrition and Metabolism, Department of Pediatrics, Cerrahpasa Medical Faculty, Istanbul University-Cerrahpasa, 34098 Istanbul, Turkey; dracaz@iuc.edu.tr (A.C.A.Z.); tanyel.zubaioglu@iuc.edu.tr (T.Z.); 3 Division of Medical Laboratory Techniques, Department of Medical Documentation and Techniques, The Vocational School of Health Services, Istanbul University-Cerrahpasa, 34295 Istanbul, Turkey; mehmet.cansever@iuc.edu.tr

**Keywords:** phenylketonuria, low-phenylalanine diet, tetrahydrobiopterin, economic damage

## Abstract

Phenylketonuria is an inherited metabolic disorder that leads to neurobehavioral dysfunction. The main treatment is a low-phenylalanine diet and/or the cofactor tetrahydrobiopterin. Regular outpatient follow-up care and measurement of the phenylalanine levels in the blood are required. We aimed to analyze the economic burden of phenylketonuria on families and the state. The patients with phenylketonuria were divided into three groups according to their treatment: a low-phenylalanine diet group (*n* = 50), a tetrahydrobiopterin group (*n* = 44), and a group taking tetrahydrobiopterin together with the diet (*n* = 25). A comparative cost analysis was carried out. The annual economic burden to the state was calculated to average EUR 18,801 ± 15,345 and was lowest in the diet group, then in the tetrahydrobiopterin group, and highest in the tetrahydrobiopterin + diet group (*p* < 0.001). Out-of-pocket costs amounted to EUR 1531 ± 1173 per year, and indirect losses averaged EUR 2125 ± 1930 per year for all families. The economic loss was significantly lower in the families taking tetrahydrobiopterin than in the other groups (*p* = 0.001). The combined use of medical nutrition and BH4 treatments has been shown to increase the economic burden on the state. Reimbursing low-protein products and increasing the number of patients eligible for financial allowances may reduce the economic burden on families.

## 1. Introduction

Phenylketonuria (PKU) is an autosomal, recessive, inherited metabolic disorder caused by phenylalanine hydroxylase (PAH) gene mutations [1]. Phenylalanine hydroxylase is a hepatic enzyme that converts phenylalanine to tyrosine with the cofactor tetrahydrobiopterin (BH4). Phenylketonuria is classified according to the severity of the hyperphenylalaninemia. Individuals with blood phenylalanine concentrations of 120–600 μmol/L before the start of treatment are classified as having mild hyperphenylalaninemia; individuals with concentrations of 600–1200 μmol/L are classified as having mild phenylketonuria; and concentrations above 1200 μmol/L denote classical phenylketonuria [2]. If the disease is not treated, it leads to irreversible neurological sequelae due to phenylalanine accumulation [3,4]. Each four-week delay in treatment has been shown to reduce IQ by four points [5]. With the national newborn screening, most patients have the opportunity to be diagnosed in the newborn period [6,7]. Turkey is the country with the highest incidence (1/6661) of phenylketonuria [8]. Phenylketonuria was the first disease for which newborn screening was performed in Turkey [9]. PKU screening was introduced in 1983 and disseminated throughout Turkey in 1994 [10].

Phenylketonuria requires special dietary treatment and follow-up from the time of diagnosis [2]. The main treatment is a low-phenylalanine diet and/or the cofactor tetrahydrobiopterin [11,12,13]. Treatment with tetrahydrobiopterin lowers blood phenylalanine levels and increases phenylalanine intolerance by increasing enzyme activity (PAH) in patients who respond to BH4. However, in some patients with phenylketonuria, phenylalanine levels may be above the upper age-appropriate limit despite guideline-compliant BH4 treatment. In these patients, BH4 treatment can be combined with nutritional therapy [11,14,15]. The intake of natural protein (e.g., meat, milk, fish, eggs, bread) is very limited during treatment. Some foods naturally low in protein, such as vegetables and fruits, can be consumed with restrictions. Low-protein formulations and special low-protein foods (bread, flour, pasta, soup, rice, meat, cheese, etc.) have been developed for patients with phenylketonuria [16]. However, these are more expensive and more difficult to obtain than normal products [17].

The main goal of treatment is to preserve neurocognitive function [18]. Micronutrient, vitamin, and mineral deficiencies should be identified during the outpatient examination [19]. Frequent hospital outpatient visits are required for monitoring blood phenylalanine levels, outpatient follow-up, and other health problems associated with PKU [11]. Follow-ups in pediatric neurology and psychiatry are required for some patients [20]. Transportation, lodging, parking, some of the examination and medication fees, and out-of-pocket expenses at the hospital place an economic burden on families. Some families pay shipping fees to purchase medications and food or to send blood tests. Parents must pay more for day care and caregivers to make sure their child’s diet is followed properly. Frequent hospital visits also result in lost productivity. Some family members have to change jobs or quit their jobs to provide the special diet and care necessary for PKU. All these direct and indirect losses cause significant economic burden to families.

Some of the examination and medication fees, blood phenylalanine level monitoring, routine biochemical testing, genetic testing, IQ screening, special education and rehabilitation, hospitalization, and diaper fees for individuals with urinary incontinence are covered by the Social Security Institution of the Republic of Turkey (SSI) [21]. Phenylalanine-free formula, low-protein milk, BH4, and treatments with large, neutral amino acids are also covered by SSI. Special low-protein foods, which are an important component of the dietary treatment, are not reimbursed. Instead, SSI provides financial support for low-protein foods in a range of EUR 5.6–14.5 per month, depending on age. When the families of children with phenylketonuria apply to the Social Insurance Institution, a “Special Needs Report for Children” is issued, which provides some benefits [22]. The report is given by the state to children with chronic diseases. They can ride public transportation for free and receive 20% discounts on all domestic flights. They receive discounts on the special consumption tax (SCT) for vehicles. Depending on the socio-economic level of the family, financial support for home care is provided each month [23,24]. 

Dietary treatment reimbursements and benefits vary widely in different countries [25,26]. Phenylketonuria requires special management from the time of diagnosis [27]. Early and regular treatment is important to prevent neurological sequelae and to integrate affected individuals into society as healthy individuals [28]. Phenylketonuria represents an economic burden for both the state and families due to management of the disease [17,26,29,30,31]. The aim of our study is to prospectively determine the economic burden on families and the state of PKU patients treated with a low-phenylalanine diet and/or BH4 treatment.

## 2. Materials and Methods

### 2.1. Study Participants

A prospective observational study was conducted between March 2020 and March 2021 at the Istanbul University-Cerrahpasa, Cerrahpasa Medical Faculty, Department of Pediatrics, Division of Nutrition and Metabolism, one of the reference centers for inherited metabolic diseases in Turkey. Patients with diagnoses of phenylketonuria were included in the study. 

### 2.2. Data Collection

#### 2.2.1. Burden on Government 

Patient records and the hospital’s electronic registration system were reviewed again, and physicians were interviewed. The costs of phe-free formulas, low-protein milk, medication, tests, healthcare visits, and rehabilitation were identified. Allowances that were provided by the government were questioned. The annual economic damage to the state was calculated.

#### 2.2.2. Burden on Families

A one-to-one questionnaire was administered to families to determine their expenditures due to phenylketonuria and sociodemographic characteristics. A prospective weekly nutrition record chart was requested in order to determine the nutrition costs. The annual economic damage to families was calculated. The expenditures of families are divided into medical and non-medical expenditures. Medical expenditures included some of the examination and medication fees; extra hours of special education and rehabilitation fees; and an extra amount for diapers, which is then reimbursed by the government. The non-medical economic burden includes special low-phe products, transportation, lodging, parking, out-of-pocket expenses at the hospital, day care, shipping fees to purchase medications, food, and sending blood tests.

### 2.3. Comparison of Patient Groups

The patients were divided into three groups according to their treatment: Group 1: low-phe diet group, Group 2: patients treated with tetrahydrobiopterin, and Group 3: patients who received both tetrahydrobiopterin and a low-phe diet. The economic burdens on families and the state were compared according to age, disease severity, treatment group, disease control, phenylalanine tolerance, and sociodemographic characteristics. The medical and non-medical expenditures of the families were compared between the three groups. Based on the average exchange rate of March 2021, EUR 1 was calculated as TRY 8.34. Wealth scores were calculated by assigning a score for each item the family owned and adding these values together. The items were listed as telephone, cell phone, washing machine, dishwasher, computer, house, summer house, and car. A score of 0–4 was considered a low score and a score of 5–8 was considered a high welfare score [32]. The disease was considered uncontrolled if more than 30% of phenylalanine levels in the previous three months had been outside the target value range [30].

### 2.4. Statistical Analysis

Categorical variables were described as percentages of the total sample size. Continuous variables with a normal distribution were expressed as means ± SDs, and others with non-normal distribution as medians. The statistical differences between the given categories were calculated using the Kruskal–Wallis test (three groups) and Mann–Whitney U test (two groups) due to non-normally distributed data. Results were considered statistically significant if *p*-values were <0.05. All statistical analyses were carried out using statistical software (SPSS, version 20.0 for Windows; SPSS, Chicago, IL, USA).

## 3. Results

### 3.1. Patient Characteristics

A total of 109 families with 119 PKU patients (47% males, 53% females) participated in the study. The median ages of the patients in Groups 1, 2, and 3 were 6.6, 2.9, and 4.5 years, respectively. The distribution of the diagnosis and treatment groups is shown in Table 1.

A total of 106 (89%) patients were diagnosed by newborn screening. The median age at diagnosis was 17.5 days (1 day–17 years), and the mean age was 168.5 days. Of the patients, 12% were diagnosed two months after birth (*n*: 14). We found that 85% of patients had normal intelligence development, while 15% of patients were mentally retarded. The disease was under control in 75% of the patients.

In 100 families, only 1 child was diagnosed with PKU; in 8 families, 2 children were diagnosed; and in 1 family, 3 children were diagnosed. The average number of members in each family was four. The average age of the mother was 34 years (23–63 years), and the average age of the father was 37 years (24–70 years). The proportion of fathers with more than 8 years of schooling was 53%, and the proportion of mothers was 47%. The mean monthly per capita income was EUR 135 ± 98 (EUR 21–800), and the median monthly income was EUR 114. Of the patients, 69 (57%) had low (1–4) wealth scores, and 39 patients had high (5–8) wealth scores. Eleven patients did not answer the questions on asset value. A total of 100 patients lived in Istanbul, and 19 patients lived outside Istanbul. Eight families had to change the city in which they lived because transportation to their hospital visits was a problem, and six families had to move to an area within the city where transportation was easier. 

### 3.2. Economic Burden of the PKU on the Government

The annual economic damage to the state was determined as EUR 18,801 ± 15,345, with a median of EUR 15,834 (EUR 352–75,953), for all patients. The annual economic burdens on the families and the state are shown in Table 2 (*p* < 0.001).

The state’s economic damage was analyzed across age groups (0–3 years, 3–12 years, and over 12 years). The economic damage caused by the 0–3 age group to the state was found to be significantly lower than the 3–12 age group (*p* = 0.006) (Table S1).

When the economic damage of the state was analyzed between patients with low (1–4) and high (5–8) wealth scores, no significant difference was found (*p* = 0.55). No significant difference was found between patients with and without controlled disease (*p* = 0.075). It was found that the economic burden of breastfed patients on the state was significantly lower compared to other patients (*p* = 0.001). Spearman’s correlation analysis evaluating phenylalanine tolerance and economic damage to the state showed a weak positive correlation (*p*: 0.022; r: 0.230). When comparing the annual economic damage to the state by diagnosis, patients with mild PKU caused significantly more damage than those with classic PKU (*p* = 0.047). Patients with mild PKU incurred a mean cost of EUR 20,129 ± 13,630, with a median of EUR 16,630, while patients with classic PKU incurred a mean of EUR 17,405 ± 16,971 and a median of EUR 10,316.

It was found that the economic damage to the state was lowest in Group 1 and highest in Group 3 (*p* < 0.001). BH4 constituted the largest portion of the state’s economic damage, followed by medical food (low-protein formula and low-protein milk) in second place and examination fees in third place for the overall group. The most important source of the state’s economic damage in patients on diets was medical food. The average annual cost of the medical food of patients on diets was EUR 6859 ± 3895, and the median was calculated as EUR 6674 (EUR 2180–20,800) (Table S2).

The main economic damage in patients using tetrahydrobiopterin was caused by BH4 treatment (*n*: 44). The mean annual economic burden for BH4 was EUR 20,177 ± 12,505, with a median of EUR 16,163 (Table S2).

The most important cause of economic damage was BH4 treatment, and the second most important cause was medical nutrition in Group 3 (*n*: 25). In this group, annual BH4 costs were calculated to average EUR 31,208 ± 13,537, with a median of EUR 32,326. Annual medical food costs were calculated to be EUR 5515 ± 2516, with a median of EUR 4719 (Table S2).

There was no significant difference between Groups 1 and 3 when comparing the economic damage to the state due to medical foods (*p* = 0.168). When the BH4 costs of Group 2 and 3 were compared, they were significantly higher in Group 3 (*p* = 0.001).

### 3.3. Economic Burden of PKU on the Families

The total economic damage to the families was compared according to the treatment group. It was found that the burden in Group 2 was significantly lower than in Group 1 and Group 3 (*p* = 0.001). There was no significant difference between Group 1 and Group 3 (Table 2). It was found that the economic damage was significantly higher in patients with classic PKU than in patients with mild PKU (*p* = 0.004). There was no significant difference between the total economic damage to the families of patients with and without controlled disease (*p* = 0.055). The economic damage to the families of patients who were breastfed was lower compared to those who were not (*p* = 0.021). For breastfed patients, the annual economic burden to the family was calculated to average EUR 1520 ± 1084, with a median of EUR 1272. There was a positive correlation between per capita income and family economic loss (*p* = 0.009; r = 0.242). Spearman’s correlation analysis for evaluating phenylalanine tolerance and the economic burden on families showed a weak negative correlation (*p* = 0.027; r = −0.222). It was found that the economic burden was higher in families with highly educated fathers than in the other group (*p* = 0.006). There was no association between the mother’s educational level and economic burden (*p* = 0.11). No significant differences were found when the family economic burden was analyzed by age group (*p* = 0.135), age at diagnosis (*p* = 0.84), mental status (*p* = 1.76), wealth score (*p* = 0.5), or gender (*p* = 0.97).

When comparing the non-medical economic burden on families by treatment group, Group 2 caused a lower economic burden than Group 1 and Group 3 (*p* = 0.001) (Table S3). There was no significant difference between Group 1 and Group 3 in this comparison. No significant difference was found when comparing families’ economic losses due to medical costs by treatment group (*p* = 0.48). The most important aspect of medical economic losses for all families was the fee for special education and rehabilitation. The annual average examination fee, which was were the same for all patients, was EUR 36 ± 23. Low-protein products were the most important cause of nonmedical economic losses for families in Group 1 and Group 3; in Group 2, it was normal food. Besides food, the most important expense was travel costs.

### 3.4. Economic Damage Caused by Food

The most used low-protein products were flour, pasta, bread, and rice. Other unclassified low-protein products made up the biggest economic burden in terms of food. This group included instant soup, noodles, yogurt substitutes, tarhana, semolina, cornflakes, French fries, crackers, cookies, wafers, cakes, cookies, chocolate, hazelnut paste, fruit juice, and flavored straws. The other expenditures were for pasta and rice, respectively, for the families of dieting patients, and pasta and meat substitutes, respectively, for the families of dieting patients using BH4 (Table S4).

No significant difference was found between the diet and BH4 + diet groups when comparing families’ economic losses due to low-protein products (*p* = 0.78). The average annual costs of low-protein products for patients were EUR 1041 ± 758 in the diet group and EUR 1156 ± 900 in the BH4 + diet group. Spending on normal foods was higher in the BH4 group than in the other two groups (*p* < 0.001), but there was no significant difference between the diet and BH4 + diet groups (Table S5).

### 3.5. Indirect Losses of Families

The average annual loss of working days for the mothers of all patients was 5.3 days, with a median of 3.5 days (2–12), and the average annual loss of working days for fathers was 21.9 days, with a median of 15 days (2–60). The average annual loss of working days for other relatives was calculated as 16.5 days, with a median of 15 days (2–60). The loss of earnings due to hospital visits constituted a major economic burden. Some parents worked shifts and adjusted their working hours to allow for hospital visits. Two mothers and seventeen fathers had needed to change jobs because they had difficulty taking time off work. Fifteen mothers stopped working when their children were diagnosed with PKU, and one father was fired because he took frequent leave. The mean annual salary loss due to days off was EUR 523 ± 320, and the median was EUR 575. The indirect loss of earnings was calculated by adding up the salary deductions for days off work, lower wages due to a job change, and wages that could no longer be earned due to dismissal. The average annual loss of earnings was EUR 2125 ± 1930, and the median value was EUR 1280.

### 3.6. Financial Support Received by Families

Financial support for low-protein foods (*n*: 40) was the most common form of support received by families, averaging EUR 154 ± 27 per year. Twelve families benefited from home care support, and the average was EUR 2128 ± 61 per year. Eleven families received financial support from their relatives. Three families benefited from the reduction in vehicle tax. 

## 4. Discussion

Phenylketonuria is the first disease to be screened for during national newborn screening in our country, and the aim is to prevent irreversible neurological sequelae by initiating appropriate treatment in the neonatal period [10]. The treatment of phenylketonuria represents an economic burden for both families and the government. To the best of our knowledge, our study is the first to examine the medical and non-medical economic burden caused by PKU to the state and families according to treatment group. There are differences from country to country in the reimbursement of phenylalanine-free formulas, low-protein foods, and BH4 therapy; these differences impact patient access to treatment resources [25]. In a study conducted in the Netherlands by Eijgelshoven et al. [31], the economic burden and time lost by families due to PKU were investigated. Similarly to our study, it was found that the most important cause of economic loss for families was low-protein products. The economic burden and time loss of families due to PKU were studied by MacDonald et al. [30]. More than 90% of the patients in this study were patients with classic PKU consuming low-phenylalanine diets. In the United Kingdom, PKU patients younger than 16 years experience less economic burden from low-protein products because phenylalanine-free formulas and low-protein products are reimbursed by the government. Travel costs to attend phenylketonuria events, excess baggage fees for special items on holiday, fees for cooking utensils, and special low-protein products were the expenses incurred by families. As in our study, the economic burden to families was found to be independent of disease control. In a study conducted by Mlčoch et al. [17] in the Czech Republic, the dietary behavior and costs of patients with hereditary metabolic disease and PKU using low-protein products were investigated. While the phenylalanine-free prescriptions of patients with phenylketonuria fell within the scope of reimbursement, the low-protein products were covered by the families. It was found that an average of EUR 130 per month (EUR 1560 per year) was spent on low-protein products. Among the low-protein products, milk, flour, pastries, and pasta caused the greatest burden. According to a study conducted by Wang et al. [29] in China, disease-related expenses in patients with classic PKU amounted to 75% of the median family income. Health insurance in China does not cover the expenses required for follow-up and treatment of the disease. Some local governments cover or provide cash reimbursement for phenylalanine-free formula for patients between 6 and 18 years of age. Expenses such as low-protein foods, examination fees, laboratory tests, medications, and transportation costs are paid by the patients themselves. None of the patients participating in the study were able to access BH4 treatment due to the high cost. The average economic damage was calculated to be EUR 4374, and it was found that most of it (58% of the total damage) was related to formulas that did not contain phenylalanine. There was a positive correlation between patient age and economic burden. In patients aged 0–4 years, there was a negative correlation between disease control and economic burden to families [29]. In the studies we cited, the mean age fell into different ranges (2.2 years [29]; 14 years [17]; 11 years [31]; 7 years [30]). The distribution of the patient population in the studies by disease severity varied. The options available in each country’s health care system also varied. Charges for phenylalanine-free formula and low-protein products varied by country. Subgroups classified according to treatment and diagnosis were not compared in these studies. For all of these reasons, the studies may have reached different conclusions regarding economic burden. 

In our study, it was calculated that the annual economic burden on the state was lowest in the diet group and highest in the BH4 + diet group (*p* < 0.001). When all patients were considered, BH4 accounted for the largest portion of the state’s economic damage, with medical food in second place and examination fees in third place. When comparing the diet and BH4 + diet groups, there was no significant difference in the economic burden on the state from medical foods (*p* = 0.168). There was no significant difference in the economic burden on families between the diet group and the BH4 + diet group (*p* < 0.9). This result indicates that administration of BH4 to patients who respond to tetrahydrobiopterin but require a specific diet does not reduce the economic burden on families or the cost of medical nutrition, which is a major government expense. The combined use of medical nutrition and BH4 treatments has been shown to increase the economic burden on the state. 

When comparing the total economic damage to families according to the treatment group, it was found that the damage was significantly lower in the BH4 group than in the diet group and the BH4 + diet group (*p* = 0.001). This suggests that low-protein products are an important contributor to the economic burden on families. When comparing the economic burden on families caused by low-protein products, no significant difference was found between the diet and BH4 + diet groups (*p* = 0.78). In our study, low-protein products were the most important cause of non-medical economic burden to families in the diet and BH4 + diet groups; in the BH4 group, it was normal foods. In addition to food, travel costs were the most important cause of non-medical economic burden. The fact that the economic burden of families living outside Istanbul was significantly higher than that of families living in Istanbul supports this. The economic burden of breastfed patients was lower for both families and the state. This was an expected result, given the lower demand for medical infant formula and low-protein products. There was a weak negative correlation between phenylalanine intolerance and economic loss to the family. It is hypothesized that, as phenylalanine intolerance decreases, economic burden increases, as the proportion of families using low-protein products increases.

It was found that the economic damage was high in families with fathers who had achieved a high educational level, but the educational level of the mother did not affect the economic damage. It was suggested that, because of the patriarchal social structure of our country, fathers take a more active role in coping with the disease. Another factor affecting this could be the increase in income as fathers work and the level of education generally increases. 

Families’ loss of labor is an important cause of economic burden. While the average annual pocket expenditure of families was EUR 1531 ± 1173, the indirect losses due to the loss of productivity were calculated as EUR 2125 ± 1930 per year. Fifteen mothers had quit their jobs to take care of their children by themselves. Twenty parents, on the other hand, had problems with their jobs and changed jobs or were laid off because they frequently took leave to deal with the illness. In the study by Macdonald et al. [30], 49% of the parents had changed jobs due to illness. 

The most important part of the financial support provided by the state to patients was home care support, the second most important was the motor vehicle tax deduction, and the third most important was food support. The median values were EUR 2158 per year, EUR 4196 per year, and EUR 164 per year, respectively. The average support for home care was EUR 2128 ± 61 per year, according to the Special Needs Report for Children, and the average expenditure of all patients’ families was calculated to be EUR 1531 ± 1173 per year. According to these calculations, home care support was sufficient to fully compensate the families’ economic losses (excluding indirect losses) (*n* = 93, 78%). However, only 10% of patients used this form of support. Eighteen families had applied for the report and had not yet received support. The rest of the patients did not apply for this support for various reasons. Some families did not know enough about this support. Some families indicated that they were hesitant to request the report because they feared that their children would be classified as disabled, while other families indicated that they felt they could adequately meet their children’s needs. The indirect burden to families was greater than the out-of-pocket costs. Forty-two families of patients experienced indirect economic losses, and the average was EUR 2125 ± 1930 per year (EUR 28–7194). Adding the indirect economic losses to the family’s out-of-pocket expenses, the average economic loss was EUR 2281 per year, and home care support was sufficient to cover the total loss of 61% of patients. Forty patients benefited from government support for low-protein products. In the diet and BH4 + diet groups, the average annual expenditure on low-protein products was EUR 1080 ± 900, and the government fee for food averaged EUR 154 ± 27. This support was sufficient to cover only 14% of the prices that families paid for low-protein products.

There were some limitations due to the method of the study. There was a significant difference when comparing the ages of the patients in the tetrahydrobiopterin group and the diet group. It was not possible to form homogeneous groups in terms of age, as the selection of patients would lead to bias. There was no significant difference between the ages of the other groups. The families’ non-medical economic losses and the economic support they received were determined using a questionnaire presented to the families. Some families were unwilling to declare their income or answer questions about asset value. The reliability of the information provided by the families may have influenced the results of this study. The families’ economic losses due to food were determined from the weekly food records. The products used by the patients and their prices may change from time to time. The cost of feeding breastfed patients and infants is not consistent throughout the year. The calculated economic loss due to nutrients may not fully reflect reality. In our study, no financial compensation was calculated for the time spent treating the disease. There are few studies in the literature on the economic damage caused by this disease. Studies employing larger patient groups could provide more precise results regarding the economic impact of the disease.

## 5. Conclusions

Phenylketonuria is a disease that can be diagnosed early through newborn screening and is treatable. However, the management and treatment of the disease constitutes an economic burden on both the state and families. According to our study, the administration of BH4 to patients who respond to tetrahydrobiopterin but require specific a diet does not reduce the economic burden on families or the cost of medical nutrition, which accounts for a large proportion of government expenditure. Government support for low-protein products was found to be sufficient to cover only 14% of the money families paid for low-protein products. The economic damage to families can be reduced by including low-protein products in the scope of reimbursement and increasing the rate of economic support provided by the state. Facilitating access to special dietary products for phenylketonuria patients can help to control the disease and prevent possible neurological sequelae. Our study also showed that loss of income and work-related problems are among the main problems faced by families. The results of this study indicate that families need more support.

## Figures and Tables

**Table 1:** Distribution of diagnosis and treatment groups.

Diagnosis	Diet	BH4	BH4 + Diet
Mild PKU, *n*	10	44	7
Classical PKU, *n*	40	0	18

**Table 2:** Annual economic burden on families and state.

		Families		State	
Treatments	(N)	Mean ± SD (per Year)	*p* Value	Mean ± SD (per Year)	*p* Value
Diet	(50)	€1680 ± 1253		€7914 ± 4930	
BH4	(44)	€1069 ± 653	0.001 *	€20,566 ± 12,465	<0.001 **
BH4 + diet	(25)	€2047 ± 1445		€37,470 ± 14,628	
All groups	(119)	€1531 ± 1173		€18,801 ± 15,345	

* represents the *p* value of the Kruskal–Wallis analysis of the economic damage to the families. ** represents the *p* value of the Kruskal–Wallis analysis of the economic damage to the state.

## Data Availability

The data are not publicly available due to privacy restrictions. The data presented in this study are available upon request from the corresponding author.

## Supplementary Materials

The following supporting information can be downloaded at: https://www.mdpi.com/article/10.3390/nu16101444/s1, Table S1: Comparison of the state’s economic damage by age groups; Table S2: Analysis of the state’s economic damage in subgroups; Table S3: Non-medical economic damage to the families (excluding food fee), Table S4: Fees of low-protein foods; Table S5: Fees of normal foods.
