# Supplementary material for: Comparison of Cost Analysis in Patients with Tetrahydrobiopterin-Responsive and Non-Responsive Phenylketonuria in Turkey
Source: Nutrients. 2024 May 10;16(10):1444. doi: 10.3390/nu16101444 (PMC11124297; doi:10.3390/nu16101444)
Supplement: Supplementary file 1 [file nutrients-16-01444-s001.zip › nutrients-2779287-supplementary.pdf]

Supplement Table S1. Comparison of the state's economic damage by age groups

Age groups	N	Mean±SD	(Median)	(Min-Max) (€/year)	p value
**1-3**	37	20177±8578	(10660)	(487-29942)	0.006
**3-12**	67	22558±16025	(20663)	(352-75953)	
**12<**	15	17829±20190	(10187)	(3410-67664)	
**Overall**	119	18801±15345	(15834)	(352-75953)	

Mean ± SD, median and min-max values are given as €/year

Supplement Table S2. Analysis of the state's economic damage in subgroups

Treatment	Values	BH4	Phe-free formula and milk	Test	Examination	Special Education and Rehabilitation	Hospitalization	Other medications	Diaper	Overall damage	p value*
Diet	Mean±SD		6859±3895	62±38	260±114	793±392	38±38	1558±3697	316±264	7914±4930	<0.001*
	Median (n)		6674 (47)	52 (50)	55 (50)	974 (6)	25 (5)	189 (7)	317 (2)	7860 (50)	
	Min-Max		872-20800	24-172	55-500	255-1229	4-100	31-9941	130-504	352-23528	
BH4	Mean±SD	20177±12505		75±43	294±106		38±32			20566±12465	
	Median (n)	16163 (44)		62 (44)	300 (44)		24 (6)			16757 (44)	
	Min-Max	3776-64652		24-170	111-412		12-116			4352-64896	
BH4 + Diet	Mean±SD	31208±13537	5515±2516	73±43	379±334	777±277	44±35			37470±14628	
	Median (n)	32326 (25)	4720 (25)	64 (25)	396 (25)	778 (2)	44 (2)			36254 (25)	
	Min-Max	11330-64652	2325-10893	24-213	103-1907	581-974	19-69			16704-75953	
Overall	Mean±SD	24174±13859	6392±3519	69±41	297±185	789±348	39±36	1558±3697	316±264	18801±15345	
	Median (n)	24244 (69)	5697 (72)	59 (119)	300 (119)	974 (8)	25 (13)	189 (7)	317 (2)	15834 (119)	
	Min-Max	3776-64652	873-20800	24-213	55-1907	255-1229	4-116	31-9941	130-504	352-75953	

Mean ± SD, median and min-max values are given as €/year.

Supplement Table S3. Non-medical economic damage to the families (exluding food fee)

Treatment		Transportation	Out-of-pocket in hospital	Parking	Shipping	Day care for patient with PKU	Day care for siblings
**Diet**	Mean±SD (n)	227±362 (50)	82±91 (46)	44±30 (27)	88±91 (7)		2158
	Median (min-max)	103 (5-2275)	53 (5-470)	45 (5-88)	58 (29-288)		2158
**BH4**	Mean±SD (n)	273±305 (42)	63±77(42)	46±36 (19)	108±7 (3)	1439±1017(2)	
	Median (min-max)	43 (10-1487)	35 (1-326)	43 (2-88)	108 (100-115)	1439 (719-1439)	
**BH4 + Diet**	Mean±SD (n)	334±455 (25)	83±141 (21)	44±35 (11)	161 (1)	2662±3561 (2)	
	Median (min-max)	146 (24-1833)	882 (2-624)	32 (3-86)	161	2662 (144-5180)	
**Overall**	Mean±SD (n)	266±364 (117)	75±98 (109)	45±32(57)	100±74 (11)	2050±2251 (4)	2158
	Median (min-max)	114 (5-2275)	41 (1-624)	45 (2-88)	86 (29-288)	1439 (144-5180)	2158

Mean ± SD, median and min-max values are given as €/year.

Supplement Table S4. Fees of low-protein foods

Low-protein food	Diet				BH4 + Diet			
	n	Mean±SD	Median	Min-max	n	Mean±SD	Median	Min-max
**Bread**	31	2±2.8	(1)	(0.1-12)	13	0.8±0.7	(0.6)	(0.2-2.6)
**Flour**	35	1.3±1	(1.2)	(0.1-4.3)	20	2.6±2.6	(1.5)	(0.4-9.6)
**Rice**	27	3±3	(2)	(0.2-11.5)	14	3.2±2.5	(2.9)	(0.7-11)
**Pasta**	35	4±3.5	(3)	(0.5-13)	19	3.4±3	(2)	(0.3-10)
**Egg**	17	1.2±1	(1)	(0.5-4)	13	1.3±1.5	(0.6)	(0.2-5)
**Meat**	26	3.1±3	(2)	(0.5-11.5)	13	4±3.7	(3)	(0.5-15)
**Other products**	44	10±8.7	(8)	(0.3-36)	23	11.7±10.7	(10)	(0.5-44)

Mean ± SD, median and min-max values are given as €/week.

Supplement Table S5. Fees of normal foods

Normal foods	Diet				BH4				BH4 + Diet			
	n	Mean±SD	Median	Min-max	n	Mean±SD	Median	Min-max	n	Mean±SD	Median	Min-max
**Vegetable**	46	2.2±1.4	(2)	(0.5-6.6)	42	1±0.6	(0.9)	(0.1-2.6)	25	1.8±0.9	(1.8)	(0.3-3.7)
**Fruit**	46	1.4±0.7	(1.2)	(0.5-3.4)	42	1±0.7	(0.9)	(0.1-3)	25	1.4±0.7	(1.3)	(0.2-3.4)
**Meat**	2	3.6±0	(3.6)	(3.6)	38	3±2.2	(2.4)	(0.6-11.7)	1	4±0	(4)	(4.2)
**Milk**	14	0.6±0.5	(0.5)	(0.06-1.7)	41	1.7±0.8	(1.6)	(0.1-4.6)	12	1.2±1.2	(0.8)	(0.1-4.3)
**Flour**	9	0.5±0.5	(0.4)	(3±1.6)	40	0.5±0.5	(0.3)	(0.1-2)	8	0.6±0.6	(0.3)	(0.1-2)
**Formula**	2	3.7±3.9	(3.7)	(1-6.5)	8	4.8±8.3	(2.5)	(0.4-25)	1	0.9±0	(0.8)	(0.9)
**Ready-to-eat**	28	0.8±0.6	(0.5)	(0.2-2.4)	40	2±1.8	(1.4)	(0.1-6)	16	1±0.9	(1.2)	(0.3-3.6)
**Other foods**	39	2±1.4	(1.4)	(0.2-5.5)	41	2±1	(1.9)	(0.3-5)	20	1.5±1.5	(1)	(0.2-5.9)

Mean ± SD, median and min-max values are given as €/week.
